# Outcomes Research on Telemedicine-Delivered Gender-Affirming Health Care for Transgender Youth Is Needed Now: A Call to Action

**DOI:** 10.1089/trgh.2021.0063

**Published:** 2023-02-08

**Authors:** Mary Kathryn Stewart, Mary Kathryn Allison, Myca S. Grant Hunthrop, Sarah Alexandra Marshall, Carol E. Cornell

**Affiliations:** ^1^ Department of Health Policy and Management, Fay W. Boozman College of Public Health, University of Arkansas for Medical Sciences, Little Rock, Arkansas, USA.; ^2^ Psychiatric Research Institute, University of Arkansas for Medical Sciences, Little Rock, Arkansas, USA.; ^3^ Arkansas Commission on Child Abuse, Rape, and Domestic Violence, University of Arkansas for Medical Sciences, Little Rock, Arkansas, USA.; ^4^ Department of Health Behavior and Health Education, Fay W. Boozman College of Public Health, University of Arkansas for Medical Sciences, Little Rock, Arkansas, USA.

**Keywords:** gender-affirming health care, gender nonconforming youth, telemedicine, transgender youth

## Abstract

This article is a call to action for outcomes research on telemedicine-delivered gender-affirming health care (GAH) for transgender youth. Transgender youth, especially rural youth, are severely underserved and face many obstacles to GAH. Telemedicine reduces access barriers for underserved populations, but telemedicine for this population can be complex. Our literature search identified only five studies exploring the use of telemedicine for GAH for transgender youth. Coronavirus disease 2019 (COVID-19)-related regulatory and reimbursement changes for telemedicine may have increased transgender youths' access to such care. Research is urgently needed to increase understanding regarding access, satisfaction, safety, and health-related outcomes of telemedicine-delivered GAH for transgender youth.

## Introduction

Transgender and gender nonconforming (trans/GNC) youth in both urban and rural areas experience higher rates of depression, suicidality, and self-harm^1^; poorer health status^2^; substance use^3^; and stigma-related violence, bullying, and harassment^4^ compared with their cisgender peers. However, well-supported trans/GNC youth have been shown to have better outcomes.^5^ Parental acceptance and support^6^ and having access to gender-affirming therapy and health care from trained providers^7^ play a major role in mental- and health-related outcomes for trans/GNC children and youth.

Accessing gender-affirming medical care can be difficult enough for trans/GNC adults due to stigma, discrimination, and many associated barriers, but for trans/GNC youth the obstacles can be greater still. Even if parents or legal guardians are affirming and willing to seek help, most clinicians lack the training and confidence to provide such care for trans/GNC youth.^8^ The decision to initiate puberty suppression with gonadotropin-releasing hormone analogues in a trans/GNC youth can be complex, and current guidelines recommend involvement of a multidisciplinary team inclusive of psychological support.^9^

Moreover, guideline-consistent provision of hormone therapy for trans/GNC adolescents <18 years is criminalized by a bill recently passed into law in Arkansas and similar bills have been considered by state legislatures across the country.^10^ Given this climate of controversy and the limited number of clinicians with appropriate training and comfort in delivering such care, solutions are needed to increase access to experienced providers for trans/GNC youth and families who currently lack such care.

Telemedicine has been promoted as an important mechanism for delivering care to underserved, especially rural, populations, including children and youth.^11^ At the same time, others have highlighted challenges with implementation at the systems level and with concerns about safety, security, and confidentiality when serving vulnerable populations.^12^ These issues raise questions about the utility of telemedicine to provide gender-affirming health care (GAH) for trans/GNC youth. Given the rapid expansion of telemedicine due to coronavirus disease 2019 (COVID-19),^13,14^ this discussion is especially timely.

## Approach and Findings

We conducted a literature search to explore the extent of research on the use of telemedicine to deliver GAH for trans/GNC youth <18 years. We focused on peer-reviewed articles published since 2010 that involved trans/GNC youth receiving telemedicine for GAH. We searched three databases (PubMed, EbscoHost, and GoogleScholar) using the term “telemedicine” and various terms identifying trans/GNC youth. We found only five articles describing research on delivery of GAH for trans/GNC youth through telemedicine.

The first article, by Sequeira et al., is a descriptive study based on surveys with 12- to 26-year-old transgender youth about their interest in telemedicine-delivered GAH, as well as their interest in GAH provided by a primary care provider.^15^ Approximately half of their 204 participants expressed interest in telemedicine GAH.^15^ This response was more common among transgender youth who reported lower levels of perceived parental support.^15^ Close to half of their participants were also interested in receiving GAH in the primary care setting, particularly if the primary care provider could receive telehealth support from a GAH specialist.^15^

The second article, by Wood et al., documented that 17% of their adolescent patients were receiving GAH by telemedicine during the first 30 days of their COVID-19 pandemic telemedicine scale-up at an academic medical center.^16^ Their publication also describes a case study of initiation of gender-affirming hormone therapy for a 16-year-old trans youth through telemedicine.^16^

The third article, by Sequeira et al., describes results of an online cross-sectional survey of 57 12- to 17-year-old gender diverse youth who had received a telemedicine visit for GAH during the COVID-19 pandemic.^17^ This study found that these patients were satisfied and comfortable with telemedicine visits for GAH during the pandemic and were willing to use telemedicine in future visits.^17^ Although most preferred that their first GAH visit be in-person, fewer preferred in-person care for their follow-up visits. Sequeira et al.'s qualitative results revealed patients' perceived benefits of telemedicine, including saving time and helping them feel safe, although there were some usability concerns, such as privacy and technological difficulties.^17^

The fourth article, by Apple et al., describes the acceptability of telehealth-delivered medical and behavioral health GAH among 21 trans/GNC youth and 38 caregivers.^18^ Most of their patients and caregivers were satisfied with telehealth-delivered medical and behavioral health GAH. To our knowledge, Sequeira et al.^17^ and Apple et al.^18^ are the only studies documenting the experiences and/or perceptions of trans/GNC youth who had actually received GAH through telemedicine.

The fifth article, by Lee et al., surveyed pediatric endocrinologists about virtual GAH services provided to trans/GNC youth before and during the COVID-19 pandemic.^19^ This study found that most (88%) respondents were not offering virtual GAH services before the pandemic, but only 8% of respondents were not offering these services during the pandemic. Although most respondents were continuing to see patients in-person, most were also offering virtual visits.^19^

## Conclusion

Although the original purpose of our search was to explore the extent of research on the use of telemedicine for GAH for trans/GNC youth, we found only five studies examining this specific type of care.^15–19^ Other studies have examined digitally delivered interventions for sexual and gender minority youth such as HIV prevention^20^ and mental health services.^21^

Stephenson et al.^20^ focused only on trans youth and found among those who chose to participate in their web-based intervention, satisfaction was high.^20^ However, participation in the intervention, which involved home-based HIV self-testing with a remote counselor present by video, was lower than among the control group, which conducted home-based HIV self-testing and reported their results online.^20^ Unfortunately, data on reasons for nonparticipation were not systematically collected.^20^

Before the COVID-19 pandemic, few Americans had received care through telemedicine,^22^ and many patients faced barriers such as age, computer or eHealth literacy, level of education, home bandwidth issues, and lack of awareness of existing telemedicine services.^23^ However, patients who live in rural areas, have prior internet use, and have higher educational attainment are more likely to be amenable to telemedicine.^22^ With the national increase in telemedicine use since the onset of the COVID-19 pandemic,^13,14^ patient hesitancy toward telemedicine may decline as patients become more familiar with the technology and more aware of virtual services.

Hamnvik et al. have published an extensive discussion of how patients' improved access to telemedicine can benefit trans/GNC patients by removing barriers related to travel, lack of locally available trained providers, and concerns about discrimination, mistreatment, and confidentiality.^24^ However, trans/GNC individuals may still face issues accessing telemedicine, such as lack of safe and confidential spaces to access virtual services, inexperience with digital technologies, and issues with reimbursement.^24^

Trans-affirming medical care for trans/GNC youth is primarily offered in urban areas where wrap-around services and specialists are more available. However, as a result of COVID-19, the Centers for Medicare and Medicaid Services, state Medicaid programs, and private payers enacted changes permitting greater reimbursement for telemedicine visits than previously allowed.^25^ Uncertainty remains regarding the durability of the regulatory and reimbursement changes that have enabled better telemedicine access during the pandemic. This increased access to telemedicine has had clear benefits, considering that most trans/GNC youth reported being satisfied and comfortable with telemedicine visits for GAH during the pandemic.^17^

Distance accessible online platforms such as Queer Doc, Queer Med, Plume, and Folx Health have medical teams that provide GAH services for trans/GNC individuals in states within their service areas. Queer Doc and Queer Med provide both puberty suppression and hormone therapy for trans/GNC youth in partnership with local primary care providers and parents. Research is needed to determine the extent to which these expanded services are improving health care access and outcomes for trans/GNC youth.

Our literature search was not an exhaustive review of the literature and our focus on a narrow definition of telemedicine clearly excluded studies of broader telehealth interventions of potential benefit to trans/GNC youth. Yet our definition was intentionally narrow because access to these specific services is so severely limited for trans/GNC youth across the United States. Indeed, the law passed this year in Arkansas, and now being challenged in court,^10^ criminalizes clinicians who provide GAH to trans/GNC youth <18 years. The implications of this law for out-of-state providers serving Arkansas minors through telemedicine are, at this point, unclear.

The lack of health care services available to address the needs of trans/GNC youth is a critical health equity concern. Telemedicine has a potential role to play in increasing access to GAH and improving outcomes for this population. We found only five studies published in the past decade exploring the use of telemedicine specifically for GAH with trans/GNC youth. This recent research suggests telemedicine for GAH is acceptable to trans/GNC youth.

**FIG. 1.**
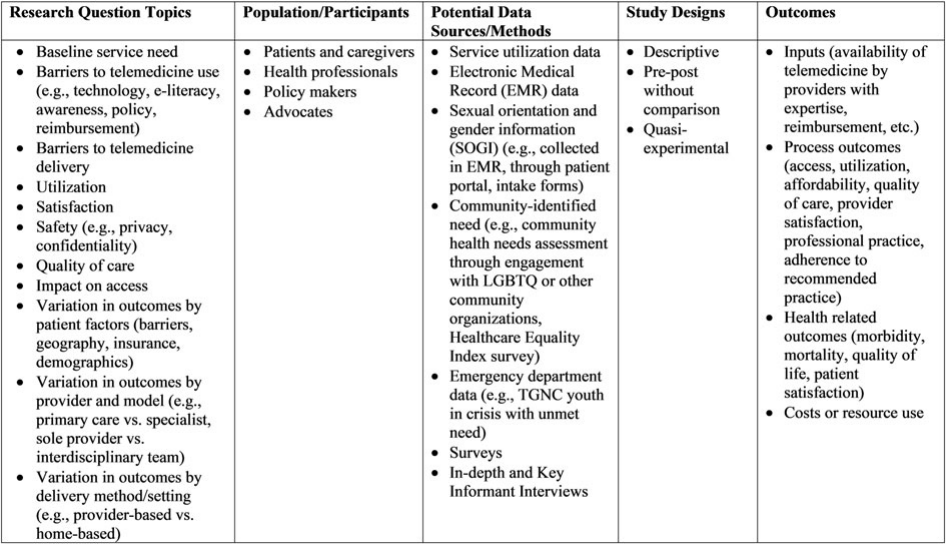
Research on telemedicine-delivered gender-affirming health care for trans/GNC youth framework of research needs.

As telemedicine use increases, research is urgently needed to document the impact of such services on trans/GNC youths' access to GAH and on their satisfaction, safety, and health-related outcomes, particularly for those who currently lack in-person access to such care (see Fig. 1 for framework of research needs). We call on providers and researchers to examine these issues and disseminate their findings about the important work they are doing to increase access to gender-affirming health care for trans/GNC youth.

## Abbreviations Used

COVID-19: coronavirus disease 2019
GAH: gender-affirming health care
trans/GNC: transgender and gender nonconforming
